# Mechanism exploration of synergistic photo-immunotherapy strategy based on a novel exosome-like nanosystem for remodeling the immune microenvironment of HCC

**DOI:** 10.1186/s40580-024-00441-6

**Published:** 2024-08-14

**Authors:** Yichi Chen, Xudong Li, Haitao Shang, Yucao Sun, Chunyue Wang, Xiaodong Wang, Huimin Tian, Huajing Yang, Lei Zhang, Liwen Deng, Kuikun Yang, Bolin Wu, Wen Cheng

**Affiliations:** 1 Department of Ultrasound, Harbin Medical University Cancer Hospital, No.150, Haping Road, Nangang District, Harbin, 150081 China; 2 Department of Breast Surgery, Harbin Medical University Cancer Hospital, Harbin, China; 3 School of Life Science and Technology, Harbin Institute of Technology, No. 92, West Dazhi Street, Nangang District, Harbin, Heilongjiang 150080 P. R. China

**Keywords:** Tumor-associated-macrophages, Exosome-like nanomedicine, Hepatocellular carcinoma, Synergistic photo-immunotherapy strategy, Single-cell RNA sequencing

## Abstract

The immunosuppressive tumor microenvironment (TME) has become a major challenge in cancer immunotherapy, with abundant tumor-associated macrophages (TAMs) playing a key role in promoting tumor immune escape by displaying an immunosuppressive (M2) phenotype. Recently, it was reported that M1 macrophage-derived nanovesicles (M1NVs) can reprogram TAMs to an anti-tumor M1 phenotype, thereby significantly alleviating the immunosuppressive TME and enhancing the anti-tumor efficacy of immunotherapy. Herein, we developed M1NVs loaded with mesoporous dopamine (MPDA) and indocyanine green (ICG), which facilitated the recruitment of M2 TAMs through synergistic photothermal and photodynamic therapy. Thereafter, M1NVs can induce M1 repolarization of TAMs, resulting in increased infiltration of cytotoxic T lymphocytes within the tumor to promote tumor regression. This study investigated the effect of phototherapy on the immune environment of liver cancer using single-cell RNA sequencing (scRNA-seq) by comparing HCC tissues before and after MPDA/ICG@M1NVs + NIR treatment. The results showed significant shifts in cell composition and gene expression, with decreases in epithelial cells, B cells, and macrophages and increases in neutrophils and myeloid cells. Additionally, gene analysis indicated a reduction in pro-inflammatory signals and immunosuppressive functions, along with enhanced B-cell function and anti-tumor immunity, downregulation of the Gtsf1 gene in the epithelial cells of the MPDA/ICG @M1NVs + NIR group, and decreased expression of the lars2 gene in immune subpopulations. Eno3 expression is reduced in M1 macrophages, whereas Clec4a3 expression is downregulated in M2 macrophages. Notably, the B cell population decreased, whereas Pou2f2 expression increased. These genes regulate cell growth, death, metabolism, and tumor environment, indicating their key role in HCC progression. This study highlights the potential for understanding cellular and molecular dynamics to improve immunotherapy.

**Graphical Abstract:**

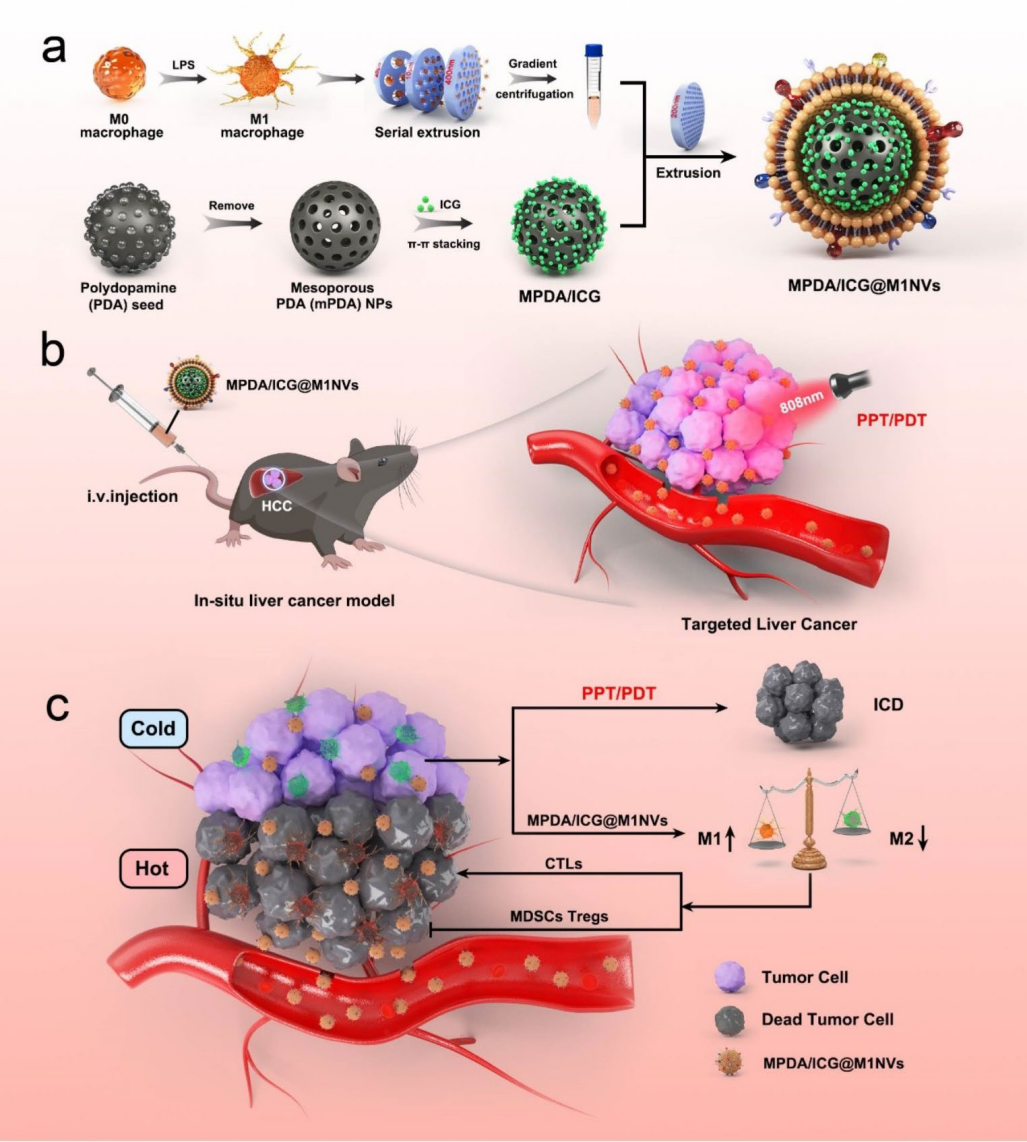

**Supplementary Information:**

The online version contains supplementary material available at 10.1186/s40580-024-00441-6.

## Introduction

Hepatocellular carcinoma (HCC) has become a leading cause of cancer death globally, with a relatively low overall 5-year survival rate (~ 18%) and a high recurrence rate after resection (~ 68%), [1] likely owing to the notable tolerance of HCC to conventional antitumor strategies, including chemotherapy, radiotherapy, and surgery [2]. Metastasis can occur in patients with HCC, even in its early stages, significantly limiting the antitumor efficacy of traditional antitumor therapies [3, 4]. In recent years, cancer immunotherapy has emerged as an effective antitumor strategy owing to its minimal side effects and long-term protection against tumor recurrence and metastasis [5–8]. Various immunotherapy strategies, including immune checkpoint blockade (ICB) and in situ immunogenic cell death (ICD) have shown encouraging antitumor effects in clinical trials [7, 9, 10]. However, the low response rate originated from the immunosuppressive tumor microenvironment (TME) significantly impedes further success of cancer immunotherapy in clinic [11, 12]. Therefore, remodeling the immunosuppressive TME represents a promising way to improve immunotherapy outcomes by restoring strong antitumor immune responses. [6, 13, 14].

As one of the most abundant immune cells in tumor lesions, tumor-associated macrophages (TAMs) are crucial for the development and immune escape of tumors by displaying an immunosuppressive (M2) phenotype [15]. Recent studies have reported that phototherapy not only initiates antitumor immune responses by inducing ICD in tumor cells, but also modulates the immunosuppressive TME by promoting the M2-to-M1 transition of TAMs [16]. However, tumor-targeted delivery of phototherapy remains a major challenge for effective attenuation of the immunosuppressive TME [17]. To improve the efficacy of phototherapy, exosomes have been explored as vehicles for tumor-targeted drug delivery [18, 19]. Notably, exosomes from TAMs have displayed outstanding tumor-targeting capabilities for the improved delivery of phototherapeutic agents. Nonetheless, the low yield of exosomes released from cells significantly restricts their further clinical application, thus driving the need for exosome-like nanocarriers for the tumor-targeted delivery of phototherapy [20].

The use of exosome-like nanocarriers marks a major advancement in tumor-targeted phototherapy delivery [21]. Their biocompatibility, targeting ability, efficient drug loading/release, and enhanced tumor penetration/retention make them ideal for phototherapeutic agents [22]. By overcoming biological barriers, reducing off-target effects, and improving therapeutic efficacy, these nanocarriers have the potential to revolutionize cancer phototherapy [23]. In the present study, we report an MPDA/ICG-coloaded exosome-like nanomedicine (MPDA/ICG@M1NVs) for synergistic tumor-targeted phototherapy and immunotherapy. Exosome-like nanoparticles were prepared by simple extrusion of M1 macrophages with MPDA/ICG encapsulated in the nanocapsules as photosensitizers and photothermal agents. The MPDA/ICG@M1NVs exhibited enhanced tumor accumulation after intravenous administration because of the tumor-homing affinity of the cell membrane and remarkable tumor growth suppression upon light irradiation owing to synergistic phototherapy and immunotherapy. Notably, exosome-like nanoparticles dramatically promote the M2-to-M1 polarization of TAMs in tumors, thus reversing the immunosuppressive TME and enhancing the antitumor efficacy of immunotherapy. Additionally, we performed single-cell RNA sequencing (scRNA-seq) to elucidate the mechanism of MPDA/ICG@M1NVs-mediated M1 polarization and provide novel insights into the antitumor performance of exosome-like nanoparticles.

## Results and discussion

### Preparation and characterization of MPDA/ICG@M1NVs

**Fig. 1:**
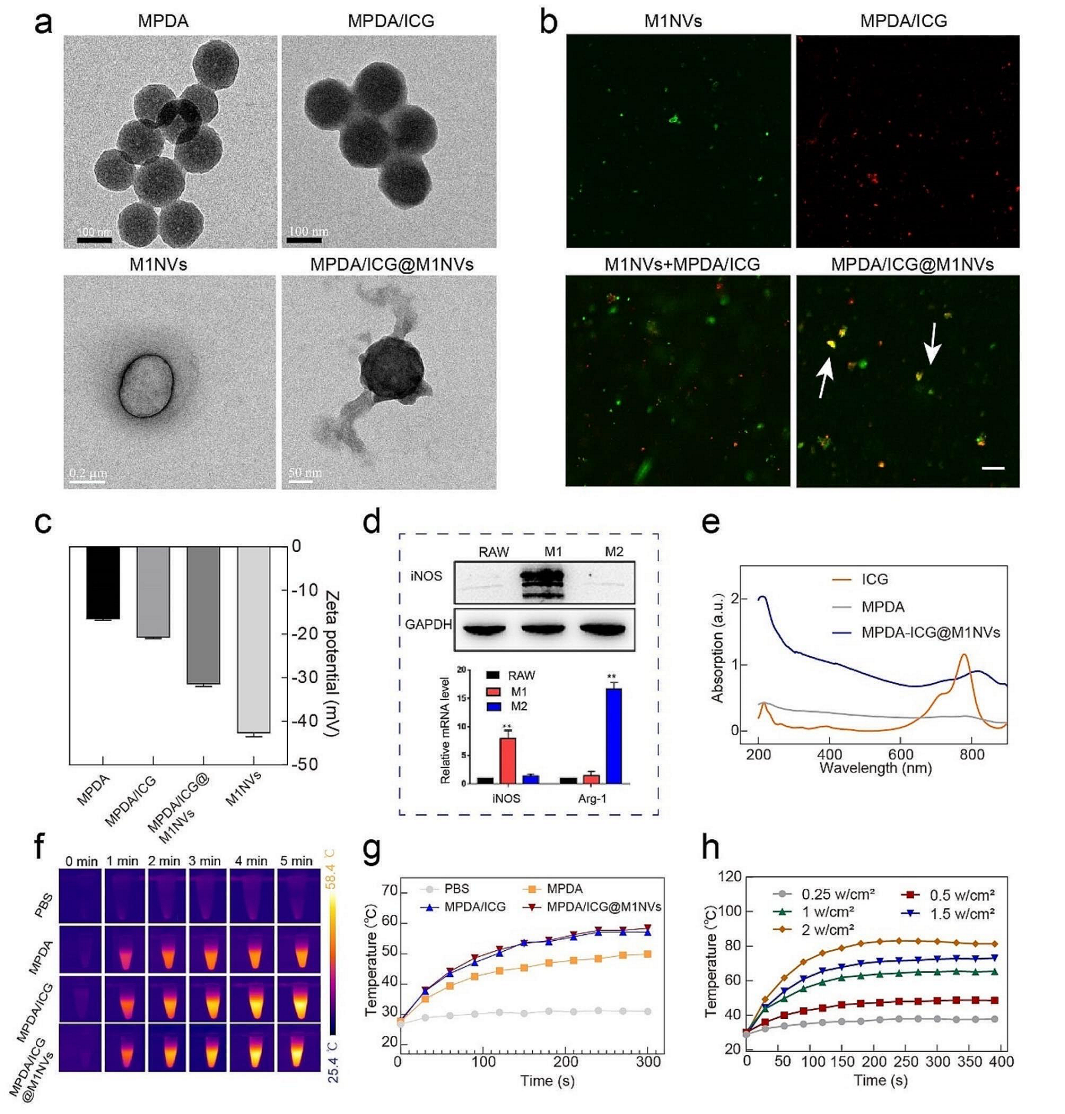
Characterization and Photothermal performance of different NPs. (**a**) TEM images of MPDA, MPDA/ICG, M1NVs and MPDA/ICG@M1NVs. (**b**) Fluorescence images of MPDA, M1NVs, a simple mixture of MPDA/ICG NPs and M1NVs and the fused MPDA/ICG@M1NVs NPs determined by fluorescence microscope; green fluorescence is from DiO and red fluorescence is from ICG, scale bar = 500 nm. (**c**) Zeta potential of MPDA, MPDA/ICG, and MPDA/ICG@M1NVs as measured by DLS. (**d**) Western blotting (WB) analyzed the protein of iNOS in M1-TAMs, and PCR analyzed the mRNA expression of iNOS and Arg-1 in M1-TAMs and M2-TAMs. (**e**) UV-vis spectra of ICG, MPDA, and MPDA/ICG@M1NVs. (**f**,** g**) Thermographic images and temperature increase profile of PBS, MPDA, MPDA/ICG, and MPDA/ICG@M1NVs solutions during NIR laser irradiation. (**h**) Temperature increase profile of MPDA/ICG@M1NVs at different NIR laser irradiation

According to scanning electron microscopy (SEM) and transmission electron microscopy (TEM), MPDA presents a uniform spherical shape with an average diameter of about 150 nm and a clear mesoporous structure, And MPDA/ICG@M1NVs with a transparent layer (~ 10 nm) of M1NV coating on the surface of MPDA/ICG NPs (Fig. 1a, Figure S1a). The hydrodynamic sizes and potentials of MPDA, MPDA/ICG and MPDA/ICG@M1NVs were determined by dynamic light scattering (DLS). The hydrodynamic diameter of the MPDA/ICG@M1NVs was 226.77 ± 6.2 nm (Figure S1b). The varied zeta potential from − 21.1 to − 32.2 mV for MPDA/ICG NPs and MPDA/ICG@M1NVs as well as the overlapping fluorescence from DiO-labelled M1NVs and ICG, respectively (Fig. 1b and c) verified successful incorporation of M1NVs onto MPDA/ICG NPs.

In addition, the increase of phosphorus on the particle surface was also observed in the SEM element mapping results (Figure S1c). The difference in the electrophoretic bands between the MPDA/ICG@M1NVs and M1NVs was also negligible, as illustrated in Figure S1d. This shows that after loading the material onto the M1NVs, the expression of the protein on the M1NVs was not affected, and the original function of the M1NVs was retained. These results further confirm the successful synthesis of MPDA/ICG@M1NVs. In addition, we verified the induction effect of M1-TAM before M1NVs preparation by WB and PCR (Fig. 1d).

UV-vis spectra and Fourier Transform infrared spectroscopy (FTIR) were used to confirm the changes in the particle structure during the synthesis of MPDA/ICG@M1NVs. The loading content of MPDA and ICG was 21.36 ± 1.04% and 53.44 ± 3.07%, respectively. The characteristic absorption of ICG was identified in the UV-Vis spectra of MPDA/ICG@M1NVs, demonstrating the successful encapsulation of ICG in M1NVs after physical coextrusion (Fig. 1e). By FTIR, it is found that MPDA carries ICG and goes through the process of coating cell membrane. Peaks at 480, 570, 665, 906, and 1103 cm^-1 indicate that both MPDA and ICG contain benzene ring structures and aromatic amine functional groups, and that these structures are still present in MPDA/ICG@M1NVs. In addition, the 1417 cm^-1 peak indicates that aniline functional groups in MPDA and ICG are also retained in MPDA/ICG@M1NVs. Therefore, it can be inferred that MPDA carries ICG and coats M1NVs, while the functional groups of MPDA and ICG do not change significantly during the whole process (Figure S1e).

### Photothermal performance of MPDA /ICG@M1NVs

Both MPDA/ICG NPs and MPDA/ICG@M1NVs induced a more rapid temperature increase than MPDA alone, indicating that exosome-like nanoparticles maintained their photothermal capacity after coating with M1NVs (Fig. 1f and g). Upon NIR irradiation, MPDA/ICG@M1NVs exhibited power density- and concentration-dependent photothermal properties as well as almost identical photothermal behaviors after four successive cycles of heating and cooling, suggesting good photothermal stability of the nanoparticles (Fig. 1h, Figure S2a-c).

### Antitumor performance of MPDA/ICG@M1NVs in Vitro

**Fig. 2:**
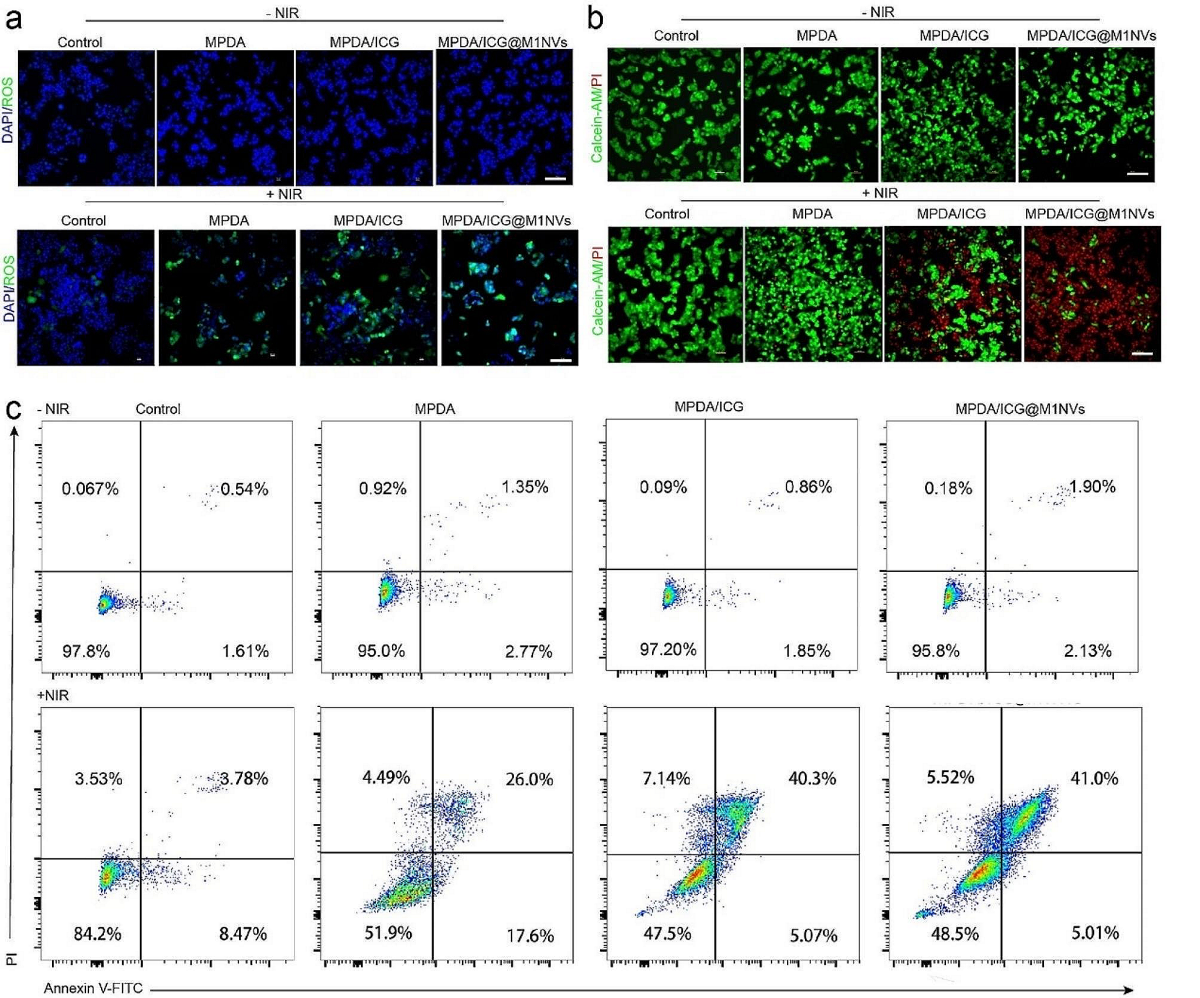
Analysis of ROS and apoptosis of synergistic photo-immunotherapy. (**a**) ROS generation in HepG2 cells in different treatment groups with or without NIR, as detected by fluorescence microscopy (scale bar = 100 μm). (**b**) Representative fluorescence images of HepG2 cells stained with calcein AM (green, live cells) and propidium iodide (red, dead cells) in different treatment groups with or without NIR, (scale bar = 100 μm). (**c**) Cell apoptosis frequency was analyzed by flow cytometry in HepG2 cells in different treatment groups with or without NIR

The uptake of MPDA/ICG@M1NVs by tumor cells was assessed prior to phototherapy. After incubation with the nanoparticles for 6 h, the fluorescence images clearly demonstrated a higher cellular uptake of MPDA/ICG@M1NVs by HepG2 cells than that of MPDA/ICG or ICG alone (Figure S3a). The cytotoxicity of various NPs was also evaluated. As illustrated in Figure S3b, MPDA/ICG@M1NVs exhibit negligible dark cytotoxicity toward normal hepatocytes (WRL68 cells) even at 300 µg/ml. In contrast, the viability of HepG2 cells declined remarkably with increasing doses of MPDA (Figure S3c), indicating that the NPs were safe after coating with M1NVs, and the MPDA/ICG@M1NVs exhibited the highest cytotoxicity on HepG2 cells under NIR irradiation. Notably, MPDA/ICG@M1NVs + NIR displayed a stronger suppressive effect on HepG2 cells than MPDA/ICG + NIR, implying a higher antitumor efficacy of M1NVs, likely owing to enhanced cellular uptake by HepG2 cells. We then analyzed the reactive oxygen species (ROS) production in the MPDA/ICG@M1NVs + NIR group and found that the introduction of light and heat promoted the photodynamic effect of ICG (Fig. 2a, Figure S3d). The PI and Calcein-AM staining assays (Fig. 2b) and flow cytometry (Fig. 2c) analysis revealed that these NPs barely induced apoptosis without NIR irradiation. In contrast, the rate of apoptosis significantly increased after NIR irradiation.

### Repolarization of M2 macrophages into M1 phenotype in vitro

**Fig. 3:**
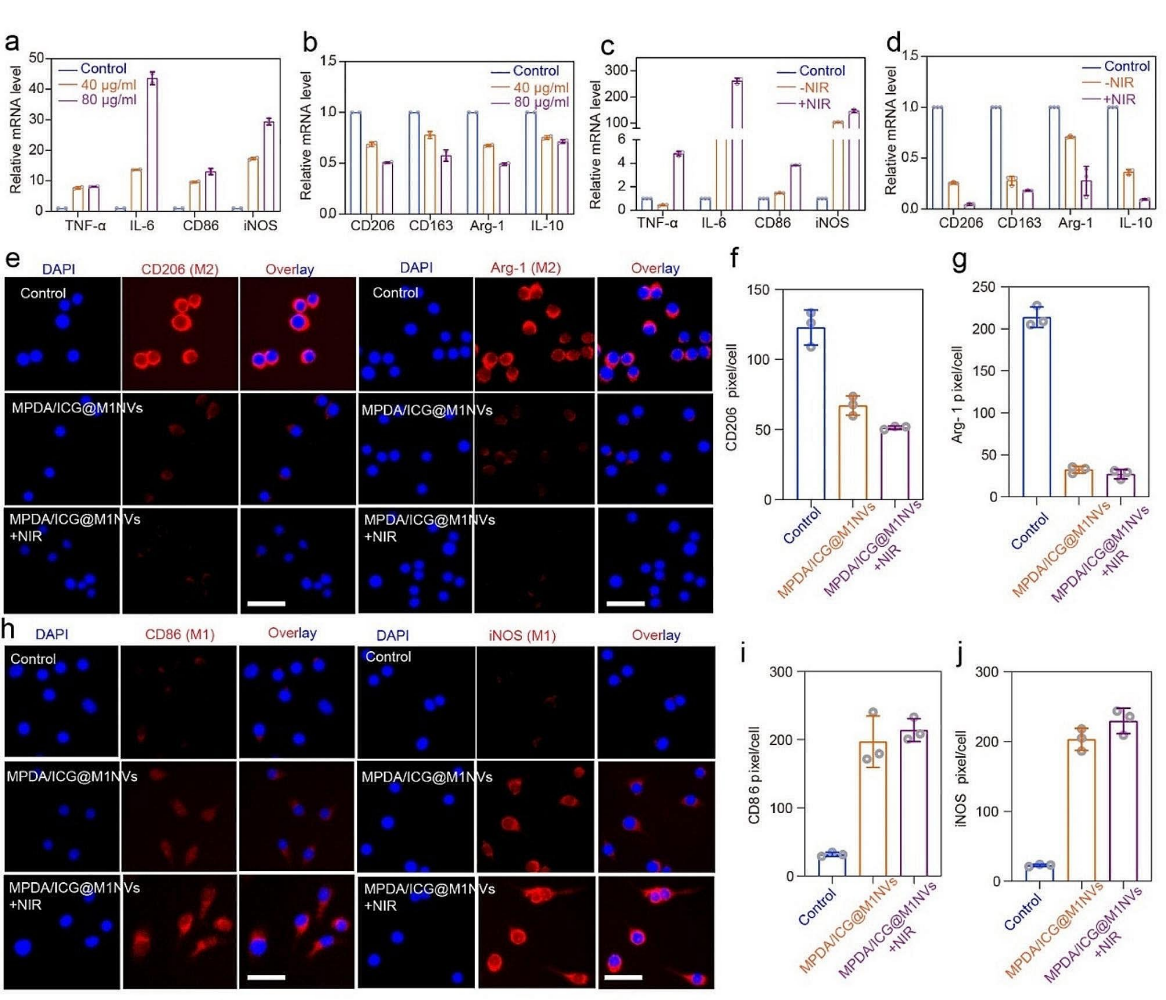
Repolarization of M2 macrophages differentiate into M1 macrophages in vitro. (**a**-**d**) Analysis of different concentrations of M1NVs to induce the differentiation of M2 macrophage into M1 macrophage. And RT-qPCR revealed the up-regulated M1 markers (TNF-a, IL-6, CD86, and iNOS) and down-regulated M2 markers (CD206, CD163, Arg-1 and IL-10) in reprogramed M2 macrophages with or without NIR. (**e**-**j**) Immunofluorescence staining and Quantitative analysis for M2 marker (CD206 and Arg-1) and M1 marker (CD86 and iNOS) in different treatment groups (scale bar = 50 μm)

We evaluated the expression of MPDA/ICG@M1NVs to determine the phenotype of TAMs, we evaluated the expression of characteristic phenotypes. Of note, the expression of M1 markers (including IL-6, TNF-α, iNOS, and CD86) in MPDA/ICG@M1NVs-treated HepG2 cells significantly increased after NIR irradiation, accompanied with dramatically decreased expression of M2 markers (CD163, CD206, IL-10, and Arg-1), indicating the M2-to-M1 transition of TAMs induced by MPDA/ICG@M1NVs plus NIR irradiation (Fig. 3a-d). This was also confirmed by immunofluorescence staining, where the fluorescence of M2 markers (CD206 and Arg-1) significantly decreased, whereas that of M1 markers (CD86 and iNOS) significantly increased in RAW cells after treatment with MPDA/ICG@M1NVs and NIR irradiation (Fig. 3e and h). Quantitative analysis of various markers also demonstrated the repolarization of M2 macrophages to the M1 phenotype mediated by MPDA/ICG@M1NVs plus NIR irradiation (Fig. 3f, g, i and j).

### Antitumor performance of MPDA/ICG@M1NVs in vivo

**Fig. 4:**
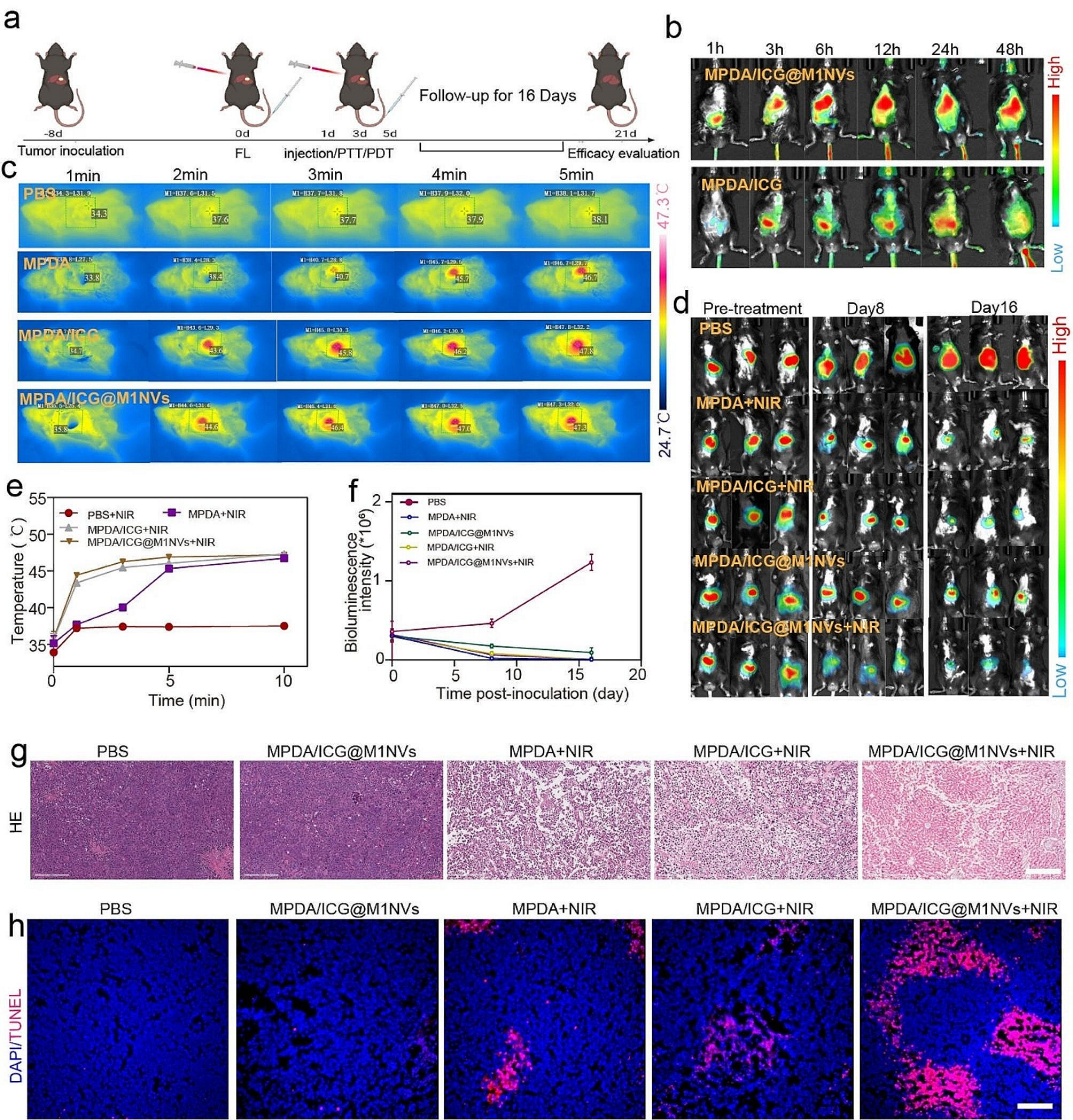
Experimental results of synergistic photo-immunotherapy in vivo. (**a**) Schematic illustration of synergistic photo-immunotherapy strategy against orthotopic HCC allograft. (**b**) Representative fluorescence images of orthotopic tumor-bearing mice at different time points after intravenous injection of MPDA/ICG and MPDA/ICG@M1NVs. (**c**) Infrared thermal images of orthotopic HCC allograft after 5 min of NIR irradiation. (**d**) Bioluminescence images of orthotopic tumor-bearing mice in different groups at the 8th and 16th days of the follow-up period. (**e**) Temperature profiles for the orthotopic HCC allograft after 5 min of NIR irradiation. (**f**) The quantitative analysis of orthotopic HCC allograft bioluminescence intensity. (**g**-**h**) H&E and TUNEL staining for pathological changes in orthotopic HCC allograft. Scale bar = 200 μm

Before evaluating the antitumor efficacy, the tumor targeting capability of MPDA/ICG@M1NVs was investigated in vivo. Notably, the fluorescence of MPDA/ICG@M1NVs emerged in the tumor region 1 h after intravenous injection of exosome-like nanoparticles. The fluorescent signals in tumors progressively increased over 6 h post-administration and were retained for 12 h, which was much stronger than the fluorescence of intravenously injected MPDA/ICG NPs, suggesting enhanced intratumoral accumulation of MPDA/ICG@M1NVs owing to their tumor-targeting properties (Fig. 4b). We then investigated the photothermal performance of MPDA/ICG@M1NVs in vivo. Infrared thermal images of orthotopic HCC allografts showed that the temperature of the tumors in MPDA/ICG@M1NVs-treated mice rose rapidly within 5 min of NIR irradiation (Fig. 4c and e). In contrast, no obvious changes in temperature were observed in the PBS-treated mice, suggesting the effective accumulation and photothermal conversion of MINVs under NIR irradiation. Encouraged by the enhanced tumor targeting and photothermal effects of MPDA/ICG@M1NVs in vivo, we further evaluated their antitumor performance in HCC tumor-bearing C57/6J mice. Tumor-bearing mice were randomly divided into five groups and intravenously injected with PBS, MPDA NPs, MPDA/ICG NPs, or MPDA/ICG@M1NVs with or without NIR irradiation (Fig. 4a). The bioluminescence of the orthotopic tumor-bearing mice in various groups was monitored on days 8 and 16 after tumor inoculation, and the bioluminescent area represented the tumor size (Fig. 4d and f). In mice treated with MPDA/ICG@M1NVs, the tumors grew rapidly within 16 d, similar to those in the PBS group. Moderate tumor growth was observed in mice exposed to MPDA and MPDA/ICG NPs with NIR irradiation. In contrast, tumor growth was significantly suppressed in mice injected with MPDA/ICG@M1NVs with NIR, suggesting enhanced antitumor efficacy of synergistic photo-immunotherapy. In addition, H&E and terminal deoxynucleotidyl transferase dUTP nick end labeling (TUNEL) staining of pathological tumor sections revealed more apoptotic cells in MPDA/ICG@M1NVs + NIR-treated mice than in all other groups, further confirming the promotion of tumor elimination by combinational photo-immunotherapy (Fig. 4g and h). No significant tissue damage was detected in the spleen, heart, kidney, or lungs in any treatment group (Figure S4a). Blood samples from the experimental animals were also analyzed. As shown in Figure S4b, routine blood and serum biochemistry analyses showed no differences among all treatment groups, indicating the superior biosafety and biocompatibility of MPDA/ICG@M1NVs. Similarly, after different treatments, the weight did not change significantly, which also verified the safety of MPDA/ICG@M1NVs (Figure S4c).

### Regulation of the immunosuppressive TME by synergistic photo-immunotherapy

**Fig. 5:**
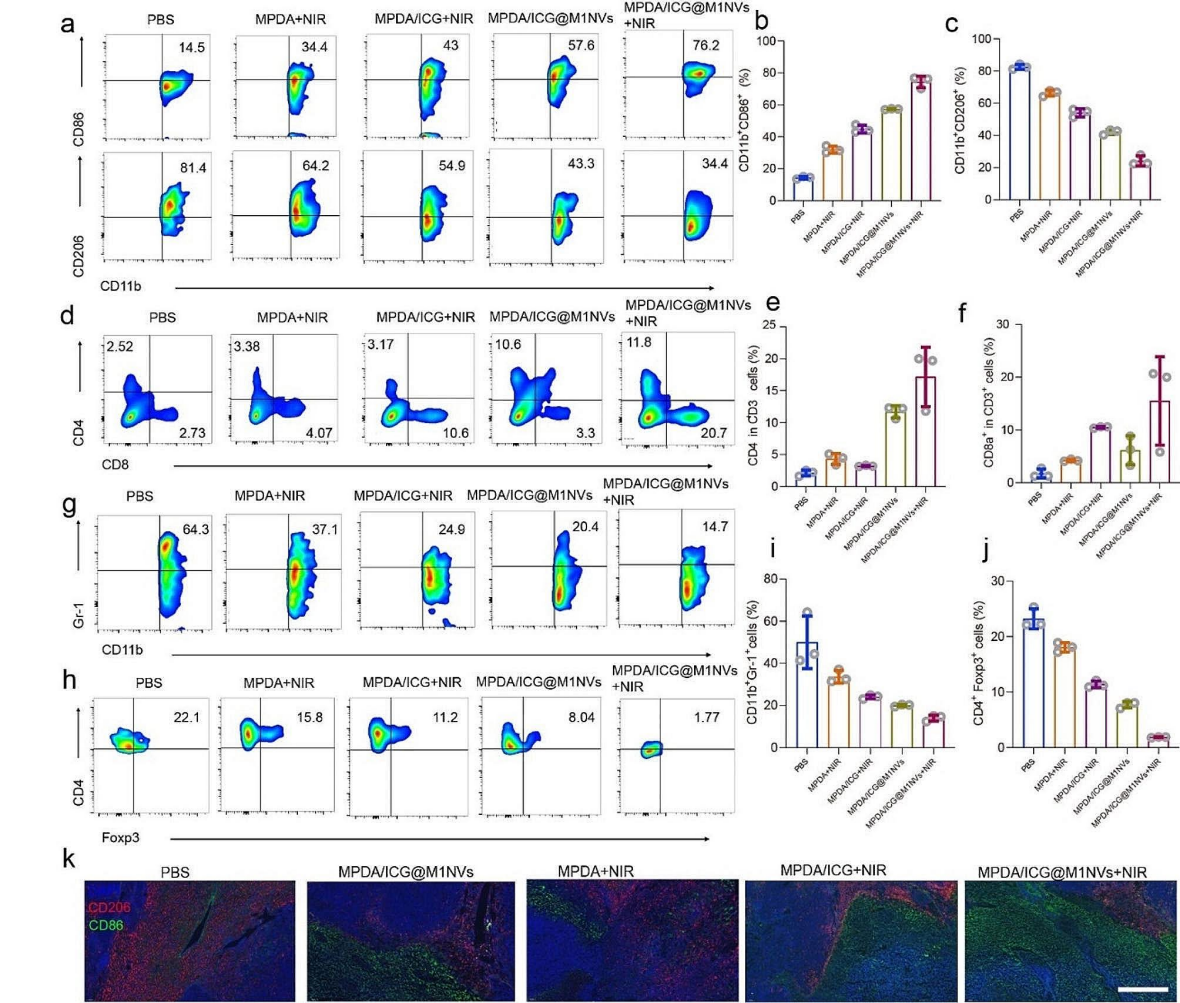
Analysis of immune cells in experimental animals after synergistic photo-immunotherapy. (**a**-**c**) Flow cytometry and quantitative analysis of M1 macrophages and M2 macrophages by CD86 marker and CD206 marker. (**d**-**f**) Flow cytometry and quantitative analysis of CD4^+^ T cells and CD8^+^ T cells. (**g**) Flow cytometry analysis of MDSCs by Gr-1 marker. (**h**) Flow cytometry analysis of Tregs by CD4 marker and Foxp3 marker. (**i**,** j**) Quantitative analysis of MDSCs (**i**) and Tregs (**j**) cells. (**k**) Representative immunofluorescence images of CD86 marker and CD206 marker expressions in the tumor tissues. Scale bars = 200 μm

To investigate the mechanism underlying the enhanced anti-tumor performance of synergistic photoimmunotherapy, tumor-infiltrated immune cells were assessed by flow cytometry. A clear M2-to-M1 transition of TAMs was observed in the tumors of mice receiving MPDA/ICG@M1NVs + NIR, as evidenced by the increased expression of CD86 and decreased expression of CD206 (Fig. 5a-c). Consequently, the population of cytotoxic CD8^+^ T cells was dramatically elevated in tumors after synergistic photo-immunotherapy (Fig. 5d-f, *p* < 0.05). In addition, immunosuppressive cells, including myeloid-derived suppressor cells (MDSCs) and regulatory T cells (Tregs), were sharply reduced in the tumors, suggesting that a strong immune response was elicited by MPDA/ICG@M1NVs + NIR (Fig. 5g-j). Immunofluorescence staining confirmed the dominant M2 TAMs in the control group and the increased M1 TAMs in mice receiving MPDA/ICG@M1NVs + NIR (Fig. 5k, red fluorescence), suggesting that the synergistic photo-immunotherapy effectively reprogramed the immunosuppressive TME by promoting the M1 polarization of TAMs, thus establishing an inflammatory antitumor niche for long term tumoricidal immune activities.

### Singlecell transcript coverage and representation

**Fig. 6:**
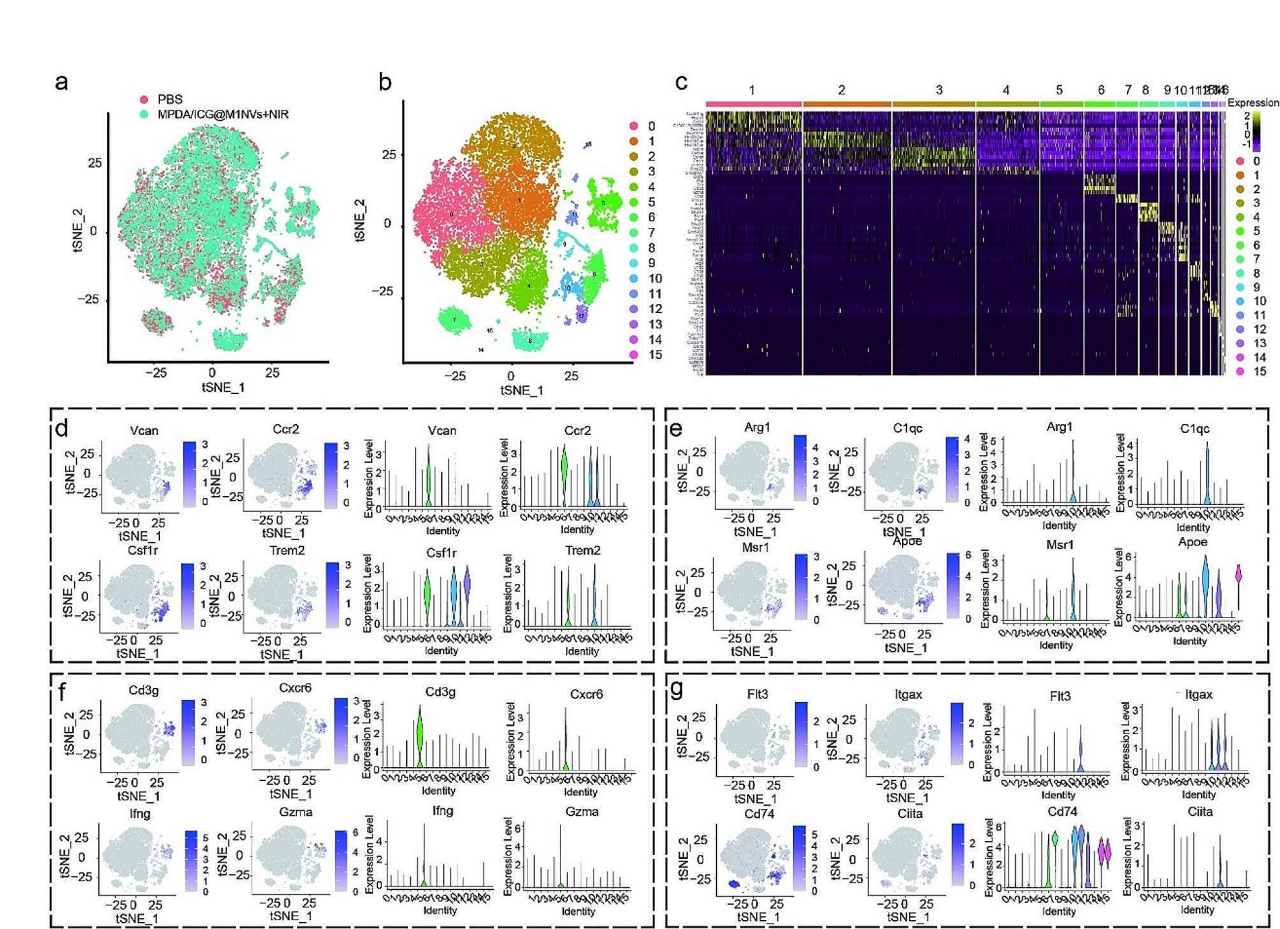
Cell heterogeneity in mouse HCC samples. (**a**) t-SNE visualized results of mouse liver cancer samples. (**b**) t-SNE visualizes cell clusters in mouse HCC samples. (**c**) Heat maps showed the top5 highly variable genes in 16 cell populations. (**d**) Annotation of M1 macrophages based on gene expression. (**e**) Annotation of M2 macrophages based on gene expression. (**f**) Annotate T cells based on gene expression. (**g**) Annotation of dendritic cells based on gene expression

Through single-cell RNA sequencing analysis, we identified 16 distinct cell subpopulations in the PBS and MPDA/ICG @M1NVs + NIR groups, with 11,290 and 9,152 cells, respectively, demonstrating significant cellular heterogeneity within the HCC tissues (Fig. 6a and b). Heatmaps were used to display the highly variable genes in each subpopulation (Fig. 6c). Cluster 6, expressing genes such as Vcan and Csf1r, represented M1 macrophages (Fig. 6d). Cells within cluster 10 were identified as M2 macrophages, primarily based on Arg1 expression (Fig. 6e). Cluster 5, expressing Cd3g and Gzma, was indicative of T cells, whereas cluster 11 was defined as dendritic cells based on the expression of genes such as Flt3 (Fig. 6f and g). Other cell types were identified based on their marker genes.

### DEGs of synergistic photo-immunotherapy

**Fig. 7:**
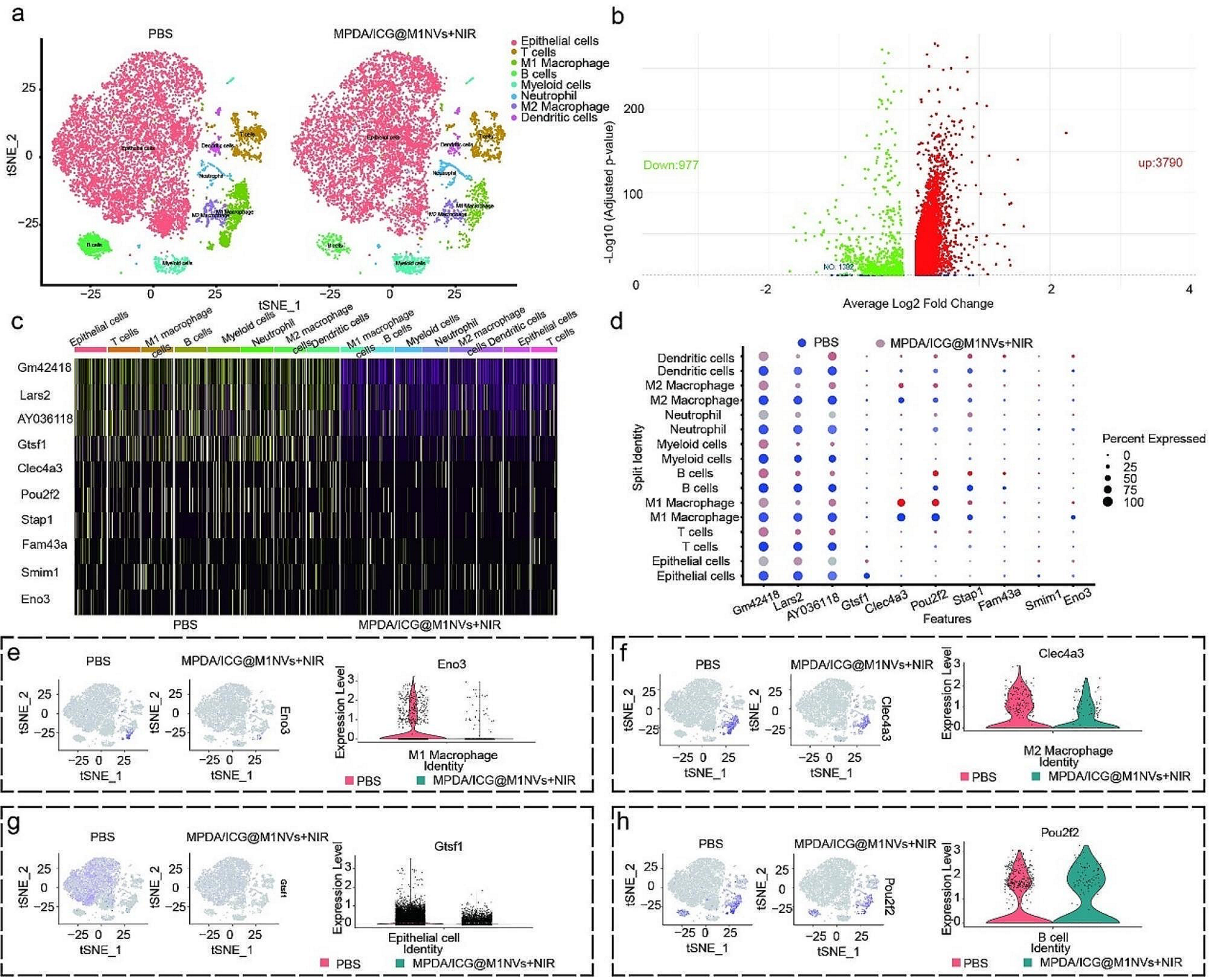
Analysis of the difference between PBS group and PDA/ICG@M1NVs + NIR group (**a**) Annotated results of t-SNE visualized grouping of mouse HCC samples. (**b**) Visualization of genetic information of mouse HCC samples with volcano map. (**c**) Heat map visualizes the expression of top10 differential genes in each cell subgroup of M2 macrophages. (**d**) The bubble map visualizes the expression difference of different genes among the top10 groups of M2 macrophages in each cell subpopulation. (**e**) Eno3 expression in M1 macrophages. (**f**) Expression of Clec4a3 in M2 macrophages. (**g**) Expression of Gtsf1 in epithelial cells. (**h**) Expression of Pou2f2 in B cells

The clusters were annotated, integrated, and renamed into eight groups based on their highly variable genes (Fig. 7a, Figure S5-S8). Post-treatment analysis revealed decreased epithelial, B-cell, macrophage, and dendritic cell populations, whereas neutrophil and myeloid cell numbers increased. The number of T cells remained largely unchanged. Differential gene expression analysis showed 4,767 genes varied significantly between the PBS and MPDA/ICG@M1NVs + NIR groups (*P* < 0.05), with 3,790 genes upregulated and 970 downregulated genes (Fig. 7b).

The differences between the PBS and MPDA/ICG@M1NVs + NIR groups were analyzed in M2 macrophages, highlighting the top 10 variably expressed genes (Fig. 7c). Bubble charts detailed these differences, noting the downregulation of the Gtsf1 gene in the epithelial cells of the MPDA/ICG @M1NVs + NIR group and decreased expression of the lars2 gene in immune subpopulations (Fig. 7d and e). Eno3 expression was reduced in M1 macrophages, whereas Clec4a3 expression was downregulated in M2 macrophages (Fig. 7f and g). Notably, the B cell population decreased, whereas Pou2f2 expression increased (Fig. 7h). These genes regulate cell growth, death, metabolism, and tumor environment, indicating their key role in HCC progression.

## Conclusions

In summary, an intelligent exosome-like nanomedicine was developed to enhance NIR-based synergistic photoimmunotherapy against HCC. The exosome-like nanoparticles significantly improved antitumor efficacy by modulating the M2-to-M1 transition of TAMs in tumors, thus attenuating the immunosuppressive TME to reinforce the antitumor immune responses elicited by the ICD of tumor cells. This study presents an innovative strategy to reshape the TME and provides novel insights into the potential clinical applications of immunotherapy in HCC. This study demonstrates the great potential of single-cell RNA sequencing technology in revealing tumor cell heterogeneity and understanding tumor pathogenesis. Our in-depth analysis of mouse HCC models provides an important molecular basis and biological insights for future HCC research and the development of therapeutic strategies.

## Methods

### Materials

Tris(hydroxymethyl)aminomethane (Tris, 99.9%), dopamine hydrochloride, Pluronic F127, 1,3,5-trimethylbenzene (TMB, 97%), Pluronic F127, and indocyanine green ICG (75%) were purchased from Aladdin Industries (Shanghai, China). The ROS assay kit, Hoechst 33,342, bicinchoninic acid (BCA) protein assay kits, and calcein AM/PI kits were purchased from Beyotime Biotechnology. DNA enzymes, collagenase, and other reagents were obtained from Sigma-Aldrich. The antibodies were purchased from BioLegend.

### Preparation of M1NVs

M1NVs were produced from M1 macrophages. In short, RAW264.7 cells (M1 macrophages) treated with LPS were suspected to be suspended in PBS at a 5 × 10^6^ cells/ml concentration. The extracellular vesicles were extruded 14 times sequentially via polycarbonate membrane filters (Whatman) with 10 μm, 1 μm, and 400 nm pore sizes through applying a mini-extruder (Avanti Polar Lipids) to achieve nanoscale extracellular vesicles. Extracellular vesicles were subsequently ultracentrifuged at 4 °C for two hours in a density gradient created by the 10% and 50% OptiPrep layers. Extracellular vesicles derived from each layer interface were subjected to further ultracentrifugation at 100,000 g for two hours at a temperature of 4 °C in order to acquire M1NVs [24, 25]. M1NVs fragments were gathered and then lyophilized for quantification and storage at -20 °C. The fragments were re-dispersed in PBS and ultrasonicated for 10 s prior to use.

### Preparation and characterization of different NPs

In short, ethanol (60 mL) was mixed with deionized water (65 mL) that contained F127 (0.36 g) and TMB solution (417 µL). After stirring for 30 min, 90 mg Tris and 60 mg dopamine hydrochloride were added to the mixture. After 24 h at ambient temperature, the crude product was collected via centrifugation. For template removal, the crude product was rinsed three times with an acetone/ethanol mixture (volume ratio of 1:2). Finally, MPDA nanoparticles were obtained. ICG was then mixed with MPDA (1 mg) in deionized water (10 ml) at an ICG/MPDA weight ratio (of 1:10) [24]. Afterwards, by shaking the mixture for 12 h, the ICG was completely loaded into the MPDA with π-π stacking action. The resulting ICG-loaded MPDA nanoparticles (MPDA/ICG) were collected via centrifugation. The remaining wash water and ICG were collected. The ICG loading effect was assessed using UV-Vis spectroscopy and subsequently mixed with the M1NVs solution and coextruded at a minimum of seven turns by weight ratio (MPDA/ICG NPs/M1NVs NPs = 1:2) through 400 and 200 nm polycarbonate membrane. Afterwards, the mixture was centrifuged at 10,000 g for ten minutes at 4 °C to yield the resulting formulation (MPDA/ICG@M1NVs).

The morphology, zeta potential, and size distribution of the MPDA/ICG@M1NVs, MPDA/ICG NPs, and MPDA NPs were assayed using TEM (JEOL, Japan), SEM (Hitachi SU8020, Japan) and a Zetasizer Nano ZS (Malvern, UK). FTIR spectroscopy was performed using an infrared spectrometer (Nicolet iS5, Thermo Fisher Scientific, USA), and UV–vis absorption spectroscopy was performed using a UV–vis spectrophotometer (SHIMADZU, Japan).

### Characterization of total protein in M1NVs and MPDA/ICG@M1NVs

The total proteins of NPs and M1NVs were characterized by SDS-PAGE. Fresh MPDA/ICG@M1NVs and M1NVs fragments were lysed using RIPA, and a BCA Protein Assay Kit (Beyotime Biotechnology) was used to determine the total protein concentration. Samples were denatured at a temperature of 100 °C for 10 min following mixing with loading buffer. SDS-PAGE electrophoresis was conducted with 20 µg of protein samples, and the obtained gels were stained in Coomassie Blue for 20 min and subsequently imaged. MPDA/ICG NPs without a membrane coating were used as controls.

### In vitro photothermal performance

The formulations of MPDA/ICG@M1NVs, MPDA/ICG NPs, and MPDA NPs with the same concentration of MPDA (75 ug/mL) were added to the EP tube. NIR irradiation (808 nm, 1 W/cm^2^) was applied to treat all samples for 8 min, and then IR imaging was performed every 30 s. To investigate the photostability of the obtained materials, 75 ug/mL of MPDA/ICG@M1NVs were irradiated via NIR irradiation (808 nm, 1 W/cm^2^) for five cycles, subsequently recording by IR imaging.

### Cell culture

H22 cells (mouse hepatocellular carcinoma) were cultivated in a 37 °C incubator with RPMI-1640 medium (10% FBS and 100 IU/mL penicillin-streptomycin). HepG2, WRL68 and RAW264.7 cell lines were cultivated in a 37 °C incubator with Dulbecco’s modified eagle medium (100 IU/mL penicillin-streptomycin with 10% FBS).

### Cellular uptake assay

To identifying the MPDA/ICG@M1NVs cellular uptake behavior in HepG2 cells, HepG2 cells were inoculated in confocal discs at a density of 1.5 × 10^5^ cells per dish. The cells were then placed in fresh medium containing free MPDA/ICG@M1NVs, MPDA/ICG, or ICG. After 4 h of incubation, the cells were washed thrice with PBS and stained with Hoechst stain. The cells were observed under an NIR scanning confocal microscope (FLUOVIEW FV1000 (OLYMPUS, Japan)) after washing extensively with PBS.

### Evaluation of in vitro anticancer activity of different NPs

WRL68 and HepG2 cells were seeded into plates (96 well) at a 1 × 10^4^ cells/well density and cultured for 24 h. The medium was removed, and the cells were treated with varying concentrations of PBS, MPDA/ICG, and MPDA. After 24 h, a CCK-8 kit was used to assay the relative viability of the cells according to the manufacturer’s instructions.

### Measurement of intracellular ROS generation

The effect of MPDA, PBS, MPDA/ICG@M1NVs and MPDA/ICG with NIR irradiation (808 nm, 1.0 W/cm^2^) on the production of ROS by HepG2 cells was investigated via the DCFH-DA ROS probe in accordance with the manufacturer’s instructions [26].

### Repolarization of M2-like macrophages into M1-like phenotype in vitro

RAW264.7, cells were grown in plates (6 well) with 3 × 10^5^ cells/well and cultivated overnight. The cells were subsequently treated with murine IL-4 (40 ng/ml) for 24 h to obtain macrophages with an M2-like phenotype. Cells were cleaned thrice with PBS and incubated with M1NVs at concentrations of 40 and 80ug/ml for 24 h to acquire repolarized cells [27]. Furthermore, qRT-PCR was implemented to identify the expression of M1 (IL-6, CD86, TNF-α and iNOS) together with M2-related genes (CD163, CD206, IL-10 and Arg-1); three samples per group were examined. M2 macrophages were treated with MPDA/ICG@M1NVs and M1NVs in the presence and absence of NIR irradiation for 24 h, followed by qRT-PCR to assay the M1- and M2-associated gene expression described above and immunocytochemistry to assess the protein expression of CD86 and CD206 macrophage markers. The cells were fixed in 4% paraformaldehyde at RT for 10 min and washed with PBS. Staining was performed with primary antibodies against CD206 and CD86. Samples were then incubated in PBS with secondary antibodies conjugated to rhodamine at RT for an hour. All samples were stained and imaged using Hoechst staining and CLSM.

### Animal models

C57BL/6 mice (male, 6 weeks old) were acquired from the Laboratory Animal Center of Southern Medical University to establish a model of orthotopic HCC. H22 hepatocellular carcinoma cell suspension (5 × 10^5^cells in PBS (25 ul)) was intraperitoneally injected into the hepatic lobe. Approximately 10 days after tumor implantation, 150 mg/kg of d-luciferin (Aladdin) was administered intraperitoneally to the mice and bound to fluorescein-labeled H22 cells. The growth of tumors and xenografts was assessed by measuring the bioluminescence of H22 cells using a small-animal optical imaging system (In-Vivo Xtreme, Bruker, Germany, emission = 460–630 nm). All animal procedures were performed in accordance with the guidelines of the Institutional Animal Care and Use Committee (IACUC) authorized by Harbin Medical University (NO.82171947).

### In vivo pharmacokinetics and distribution of MPDA/ICG@M1NVs

For in vivo imaging, 200 µL of MPDA/ICG@M1NVs or MPDA/ICG were later administered intravenously in PBS at the identical concentration of ICG to H22 tumor-carrying C57BL/6 mice (*n* = 3), respectively. All mice were gastically anesthetized, and fluorescence imaging in vivo (with an emission/excitation of 821/785 nm) was documented with a small animal imaging system 1, 3, 6, 9, 12, 24, and 48 h later.

### In vivo tumor growth inhibition and safety analyses

Mice with comparable bioluminescence values of orthotopic HCC allograft tumors were randomly allocated into six groups of 10 mice each, which were intravenously administered MPDA, PBS, MPDA/ICG@M1NVs, or MPDA/ICG (the concentration of MPDA was 1 mg/kg per mouse). 24 h later, the mice were anesthetized, and the tumors were exposed to 1 W/cm^2^ of 808 nm NIR irradiation for 10 min. IR thermal images and changes in temperature were recorded using an infrared thermographic camera (FLIR One Pro, USA). After various treatments, tumor progression was assessed using histological examination and bioluminescence imaging to assess treatment efficacy. The vital signs and body weights of all the mice were determined during the 16-day follow-up period. All mice were euthanized 16 days later and serum samples were isolated. Biochemical parameters, which included Glutamyl transpeptidase (GGT), alanine aminotransferase (ALT), creatinine (CRE), aspartate aminotransferase (AST) and blood urea nitrogen (UREA), as well as total bilirubin (TBIL), were automatically analyzed through Poincare M3. The liver, spleen, kidneys, lungs, and heart were collected for hematoxylin and eosin staining.

### Tumor tissue assessments

To compare the mRNA expression of CD80 and CD206 in tumor tissues, the tumors were removed from euthanized mice 1 d after the final injection, ground with a scalpel, and homogenized. Samples of tumors were immediately frozen in the liquid nitrogen and subsequently sectioned at 4 μm thickness for TUNEL staining and immunohistochemistry. In the tissue sections, apoptotic cells were identified by applying the dUTP-digoxigenin nick-end labeling TUNEL technique (Beyotime) mediated by terminal deoxynucleotidyl transferase (TdT), followed by a TUNEL assay according to the manufacturer’s instructions. Immunohistochemical staining was performed for the M2 and M1 markers. The treatment of sections was carried out utilizing the sodium citrate buffer (0.05% Tween 20 mM and 10 mM Sodium citrate, with a pH of 6.0) at 85 °C for 10 min to allow antigen retrieval after the hydration. Tissue sections on the slides were blocked via 5% (v/v) normal goat serum (Gibco) prior to staining and were inoculated through primary antibodies to CD206 (Abcam) and CD86 (BioLegend) at 4 °C for 18 h. PBS was employed to clean the sections, which were later inoculated utilizing secondary antibodies conjugated with rhodamine for 1 h. After cleaning in PBS, the sections were mounted using a mounting medium (Vecta Mount Mounting Medium, Vector Labs Inc., Burlingame, CA, USA).

### Analysis of immune cells in TME

A single-cell suspension was prepared from the tumor tissue, and immunofluorescence staining was followed by quantitative analysis of tumor-infiltrating lymphocytes by flow cytometry. Briefly, tumor tissue was obtained from the mice euthanized one day after the final injection, which was cut into small pieces using a razor blade, and enzymatically digested through collagenase IV (1 mg/ml) DNase (0.1 mg/ml) at 37 °C for an hour. Cells were collected, stained with a cocktail of fluorescently labeled antibodies, and analyzed using a FACS Aria II (BD Biosciences). CD3-positive T cells were initially identified and screened for expression of CD8 and CD4. The following monoclonal antibodies were used: APC anti-mouse CD4, FITC anti-mouse CD3, phycoerythrin (PE) anti-mouse CD8a, and PE anti-mouse FOXP3 (BioLegends).

### Single‑cell RNA sequencing analysis

Three tumor-bearing mice in PBS and MPDA/ICG@M1NVs + NIR groups were euthanized, and tumor samples were collected, dissected, and assayed using scRNA-seq on day 16.

#### Single-cell sample prep and sequencing

##### Cell preparation

The general protocols and optimal practices for cleaning, enumerating, and concentrating cells in limited and large cell suspensions (total cell counts exceed or below 100,000, respectively) was presented with 10x Genomics^®^ Cell Preparation Guide, to prepare for usage in the 10x Genomics Single Cell Protocol.

##### Single-cell RNA sequencing

Cell suspensions were loaded onto the Chromium microfluidic chip with 3’v3 chemistry, which were later barcoded through employing a 10X Chromium controller (10X Genomics). RNA from the cells barcoded was later reverse transcribed and the sequencing library was prepared via utilizing reagents from the Chromium Single Cell 3’ v3 kit (10X Genomics) as per the instructions of manufacturer. Sequencing was performed using an Illumina (HiSeq 2000) as to the manufacturer’s instructions (Illumina).

### Data analysis

#### Quality control

Fastp was used for basic statistical analysis of raw read quality. Generally, cellRanger counts support the FASTQ file of the original base call (BCL) file produced by the Illumina sequencer as an input file. 10x Genomics^®^ does not suggest extra sequence processing.

If the clean reads are integral, the raw read sequences resulting from the Illumina pipeline in FASTQ format are pretreated using Trimmomatic software and can be summed up as follows:

1. Removal of low-quality reads: The reads were scanned using a sliding window 4 bases wide and cut off when the mean mass of each base dropped below 10 (SLIDINGWINDOW: 4:10).
2. Removal of trailing low-quality or N bases (lower than quality 3) (TRAILING:3).
3. Aptamer removal: There are two methods for removing aptamer sequences: (a) Alignment with the aptamer sequence with a matching base number > 7 and mismatch = 2; (b) When the overlapping base score of read1 and read2 were > 30, the non-overlapping region was removed (ILLUMINACLIP: adapter.fa: 2:30:7).
4. Reads less than 26 bases in length were discarded.
5. Those reads that did not create matches were excluded. The remaining reads that passed all filtering steps were considered clean reads and all subsequent analyses were performed on this basis. Finally, fastp was applied for the basic statistics on the clean reads.

### Generation and analysis of single-cell transcriptomes

Raw reads were demultiplexed and passed through a 10X filter. The default parameters were used in the Genomics Cell Ranger pipeline (https://support.10xgenomics.com/single-cell-geneexpression/software/pipelines/latest/what-is-cell-ranger). Unless otherwise mentioned, all downstream single-cell analyses were performed using Seurat and Cell Ranger [28, 29]. For each cell barcode (filtered using CellRanger) and gene, distinct molecular identifiers were calculated to construct a digital expression matrix. Secondary filtering was performed using Seurat; a gene expressed in exceed 3 cells was deemed to be expressed, requiring a minimum of 200 expressed genes per cell. The foreign cells were filtered out.

### Secondary analysis of gene expression

The Seurat package was used for normalization, dimensionality reduction, clustering, and differential data expression. We comprehensively analyzed the dataset using the Seurat alignment approach and classical correlation analysis (CCA) [30]. To perform clustering, we chose highly variable genes and created a graph that was segmented at a 0.6 resolution utilizing the principal components based on these genes. Differential expression analysis among the samples was performed using the edgeR package to derive partition-specific marker genes in accordance with the Seurat-filtered gene expression matrix [31].

### Statistical analysis

The statistical tests applied and the number of independent and replicate experiments are presented in the figure and text legends. All figures shown with error bars report mean ± s.e.m. values, except where noted. Repeated-measures ANOVA was used to identify the significance of differences between groups. Basic statistical analyses and plotting were performed using GraphPad PRISM 8.0.

### Electronic supplementary material

Below is the link to the electronic supplementary material.

Supplementary Material 1

Supplementary Material 2

## Data Availability

The scRNA-seq datasets used and analyzed during the current study were uploaded and deposited in NCBI’s Sequence Read Archive (PRJNA869679). All data or resources used in the paper are available by reasonable requirements to the leading correspondence, Prof. Wen Cheng (chengwen@hrbmu.edu.cn).

## Abbreviations

HCC: Hepatocellular carcinoma
PDT: Photodynamic therapy
PTT: Photothermal therapy
TAMs: Tumor-associated macrophages
ROS: Reactive oxygen species
scRNA-seq: Single-cell RNA sequencing
