# Supplementary material for: Mechanism exploration of synergistic photo-immunotherapy strategy based on a novel exosome-like nanosystem for remodeling the immune microenvironment of HCC
Source: Nano Converg. 2024 Aug 14;11:31. doi: 10.1186/s40580-024-00441-6 (PMC11324638; doi:10.1186/s40580-024-00441-6)
Supplement: Supplementary file 1 — Supplementary Material 1 [file 40580_2024_441_MOESM1_ESM.docx]

Supporting Information for

**Mechanism exploration of synergistic photo-immunotherapy strategy based on a novel exosome-like nanosystem for remodeling the immune microenvironment of HCC**

Yichi Chen^1, #^, Xudong Li^2, #^, Haitao Shang^1^, Yucao Sun^1^, Chunyue Wang^1^, Xiaodong Wang^1^, Huimin Tian^1^, Huajing Yang^1^, Lei Zhang^1^, Liwen Deng^1^, Kuikun Yang^3,^ ***, Bolin Wu^1,^ **, Wen Cheng^1,^ *

1. Department of Ultrasound, Harbin Medical University Cancer Hospital, Harbin, China.

2. Department of Breast Surgery, Harbin Medical University Cancer Hospital, Harbin, China.

3. School of Life Science and Technology, Harbin Institute of Technology, Harbin, Heilongjiang, 150080, P. R. China.

# * First Corresponding Author:

Full name: Wen Cheng

Department: Department of Ultrasound,

University/Hospital: Harbin Medical University Cancer Hospital

Street Name & Number: No.150, Haping Road, Nangang District

City, Country, Postal code: Harbin, China, 150081.

Tel: +86 13313677182

E-mail: chengwen@hrbmu.edu.cn

****Second Corresponding Author:**

Full name: Bolin Wu

Department: Department of Ultrasound,

University/Hospital: Harbin Medical University Cancer Hospital

Street Name & Number: No.150, Haping Road, Nangang District

City, State, Country, Postal code: Harbin, China, 150081.

Tel: +86 15663615088

E-mail: wubolin@hrbmu.edu.cn

*****Third Corresponding Author:**

Full name: Kuikun Yang

Department: School of Life Science and Technology,

University/Hospital: Harbin Institute of Technology,

Street Name & Number: No. 92, West Dazhi Street, Nangang District

City, State, Country, Postal code: Harbin, China, 150080.

E-mail: [yangkuikun@hit.edu.cn](mailto:yangkuikun@hit.edu.cn)


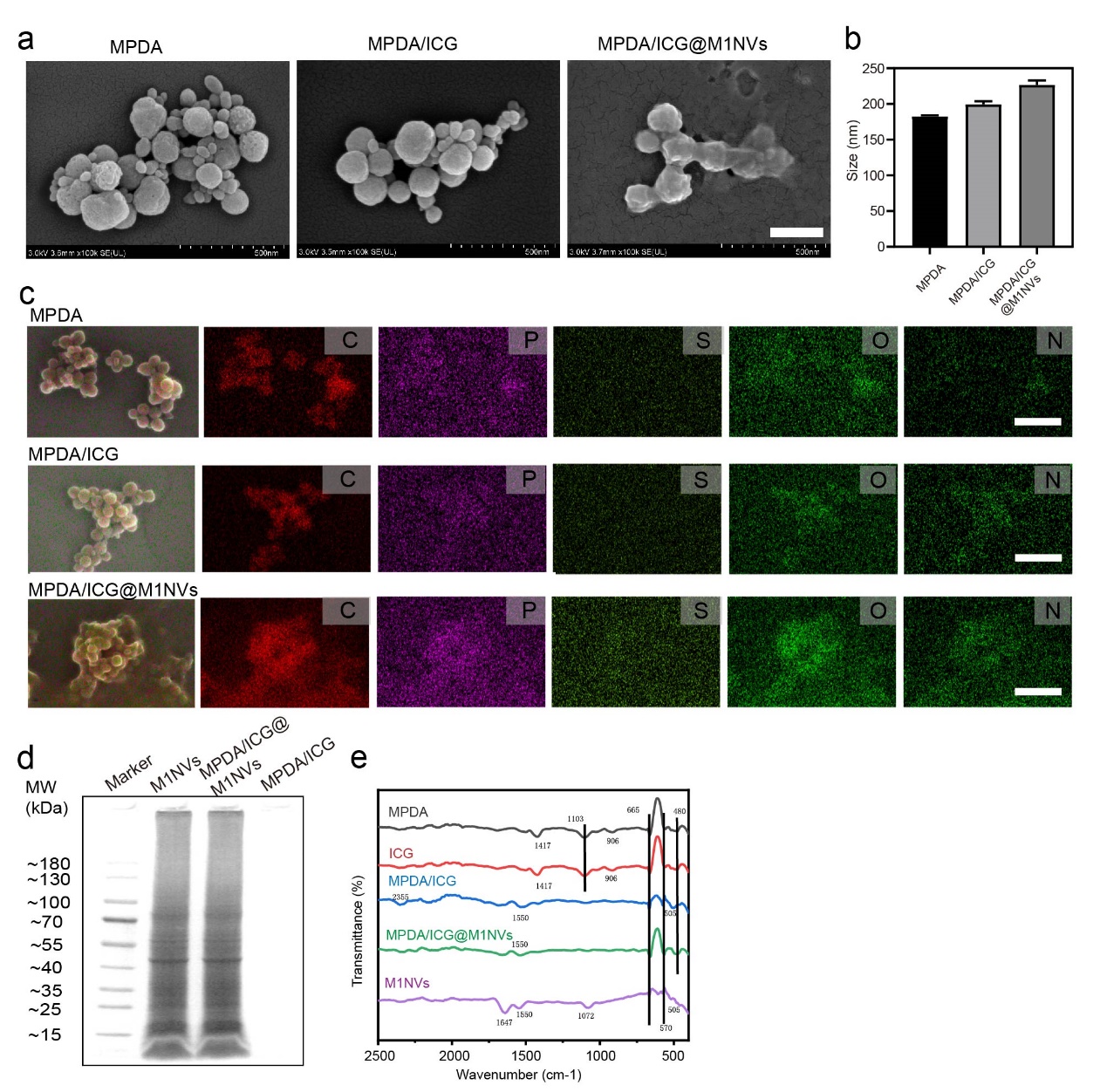


**Figure S1. Additional characterization of MPDA/ICG@M1NVs.** (**a**) SEM image of MPDA, MPDA/ICG, and MPDA/ICG@M1NVs (scale bar: 250 nm). (**b**) Particle size of MPDA, MPDA/ICG, and MPDA/ICG@M1NVs as measured by DLS. (**c**) Elemental mapping images of MPDA, MPDA/ICG, and MPDA/ICG@M1NVs (scale bar: 500 nm). (**d**) SDS-PAGE analysis of total proteins extracted from M1NVs, MPDA/ICG, and MPDA/ICG@M1NVs. MW, molecular weight. (**e**) Fourier transform infrared (FTIR) spectroscopy of MPDA/ICG@M1NVs.


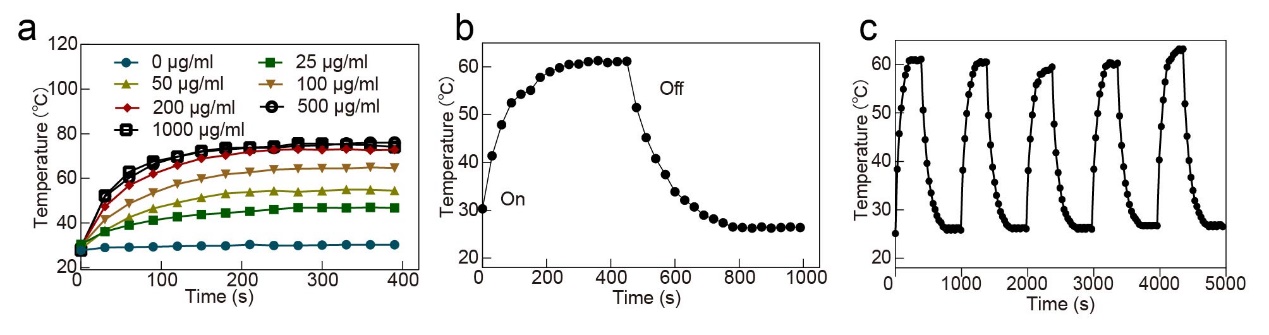


**Figure S2. Additional photothermal performance of MPDA/ICG@M1NVs**. (**a**) Temperature increase profile of MPDA/ICG@M1NVs at different MPDA concentrations. (**b**) Temperature profile of MPDA/ICG@M1NVs under photothermal heating and natural cooling. (**c**) Temperature changes of MPDA/ICG@M1NVs over five cycles of repeated NIR laser irradiation.

**
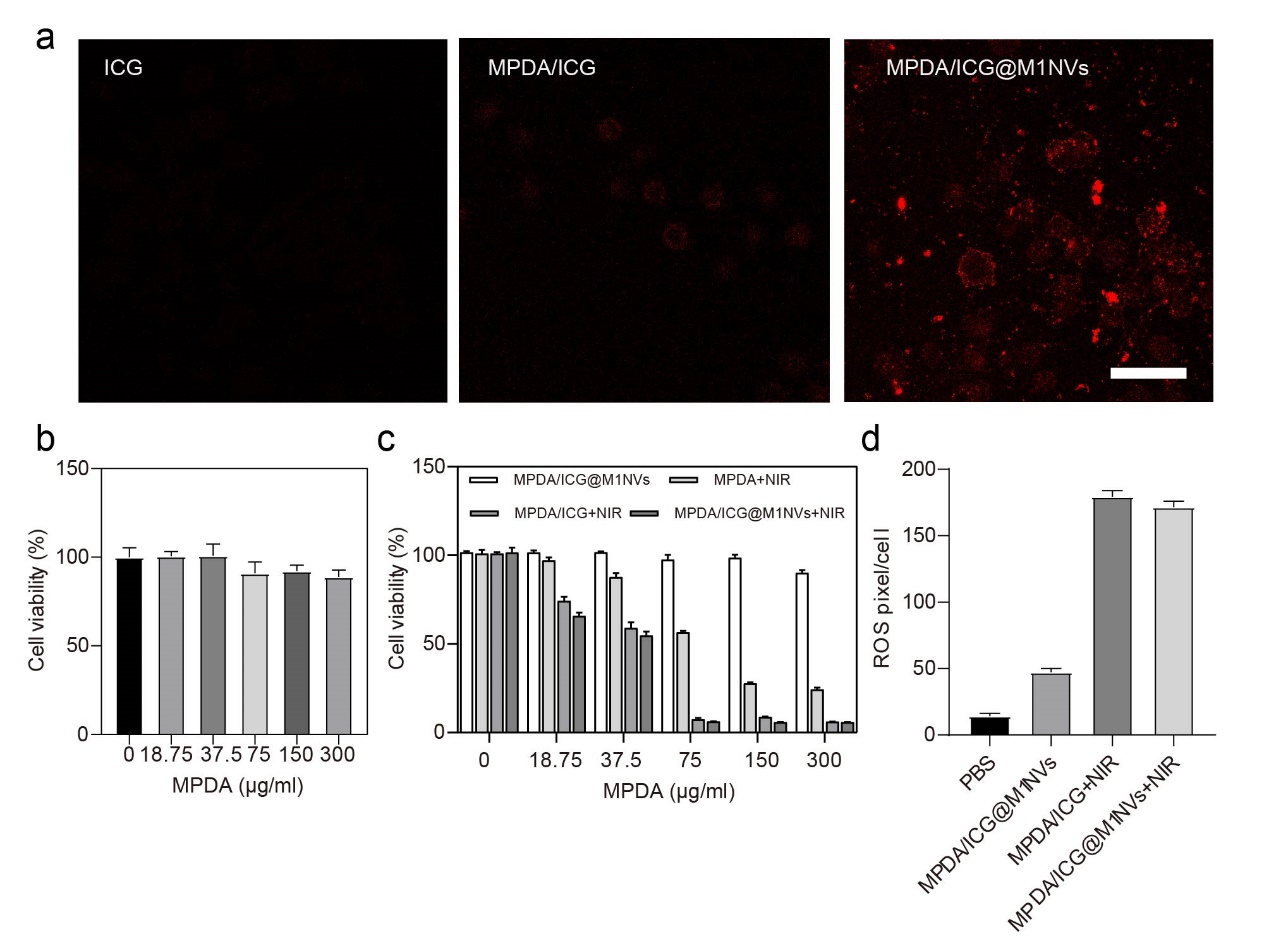
**

**Figure S3. Cell uptake and cytotoxicity of different NPs.** (**a**) Cellular uptake of the different formulations on HepG2 cells after 6 h incubation (scale bar = 50 μm) (**b**) Viability of WRL68 cells incubated with MPDA/ICG@M1NVs NPs containing different MPDA concentrations without NIR irradiation, n=3. (**c**) Viability of HepG2 cells incubated with MPDA, MPDA/ICG and MPDA/ICG@M1NVs with or without NIR irradiation, n=3. (**d**) Quantitative analysis of ROS of the different formulations.


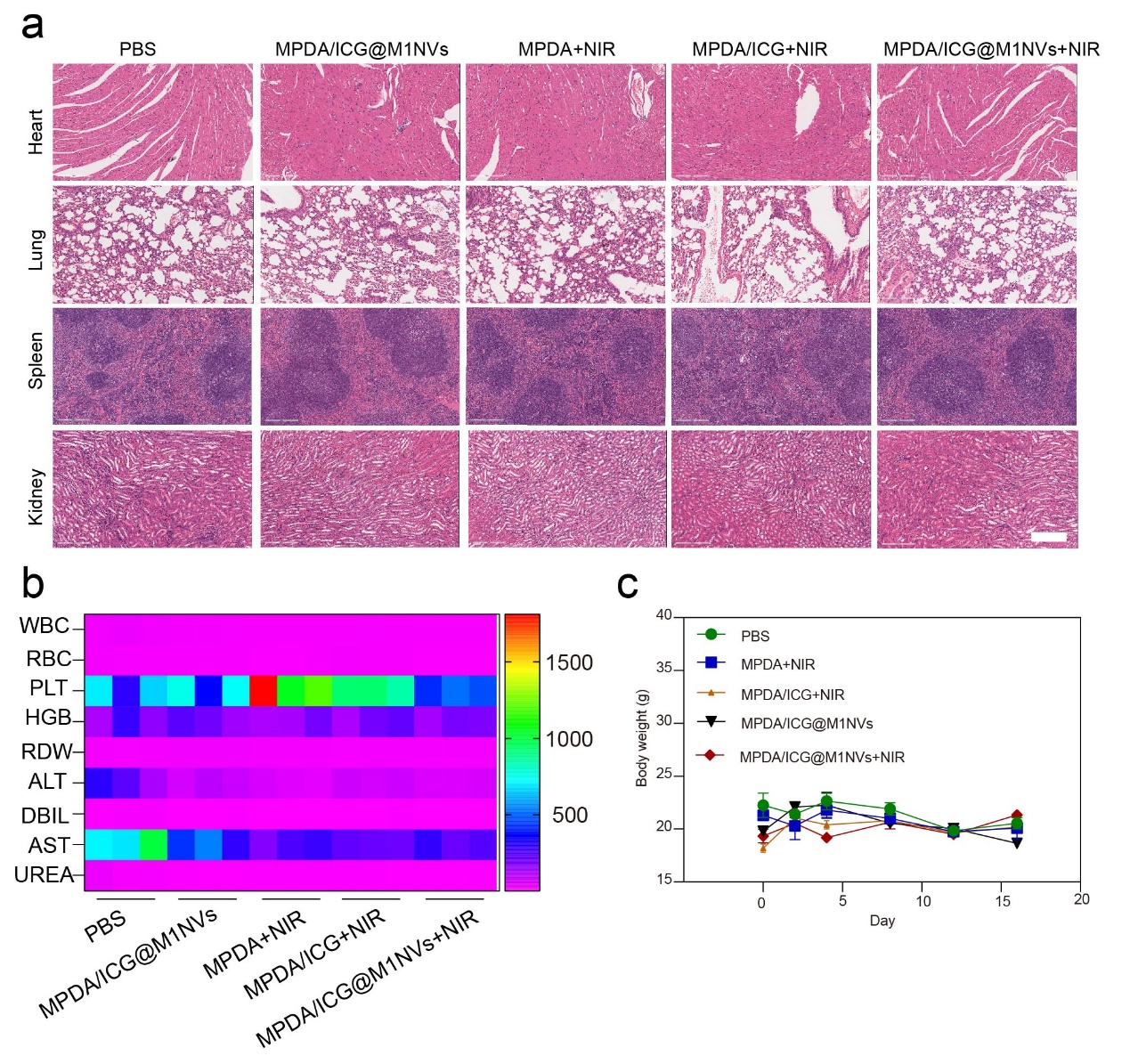


**Figure S4. Security analysis of MPDA/ICG@M1NVs.** (**a**) H&E staining of heart, spleen, lung and kidney tissue slices of experimental mice after treatment for 16 days. Scale bar: 200 μm. (**b**) Heat map of routine blood and serum biochemistry analysis of C57/6J mice after intravenous injection of different NPs. (**c**) Body weight of mice bearing H22/luc tumour during the 16 days of treatment (n = 3 per group; data are presented as the mean ± s.d.).


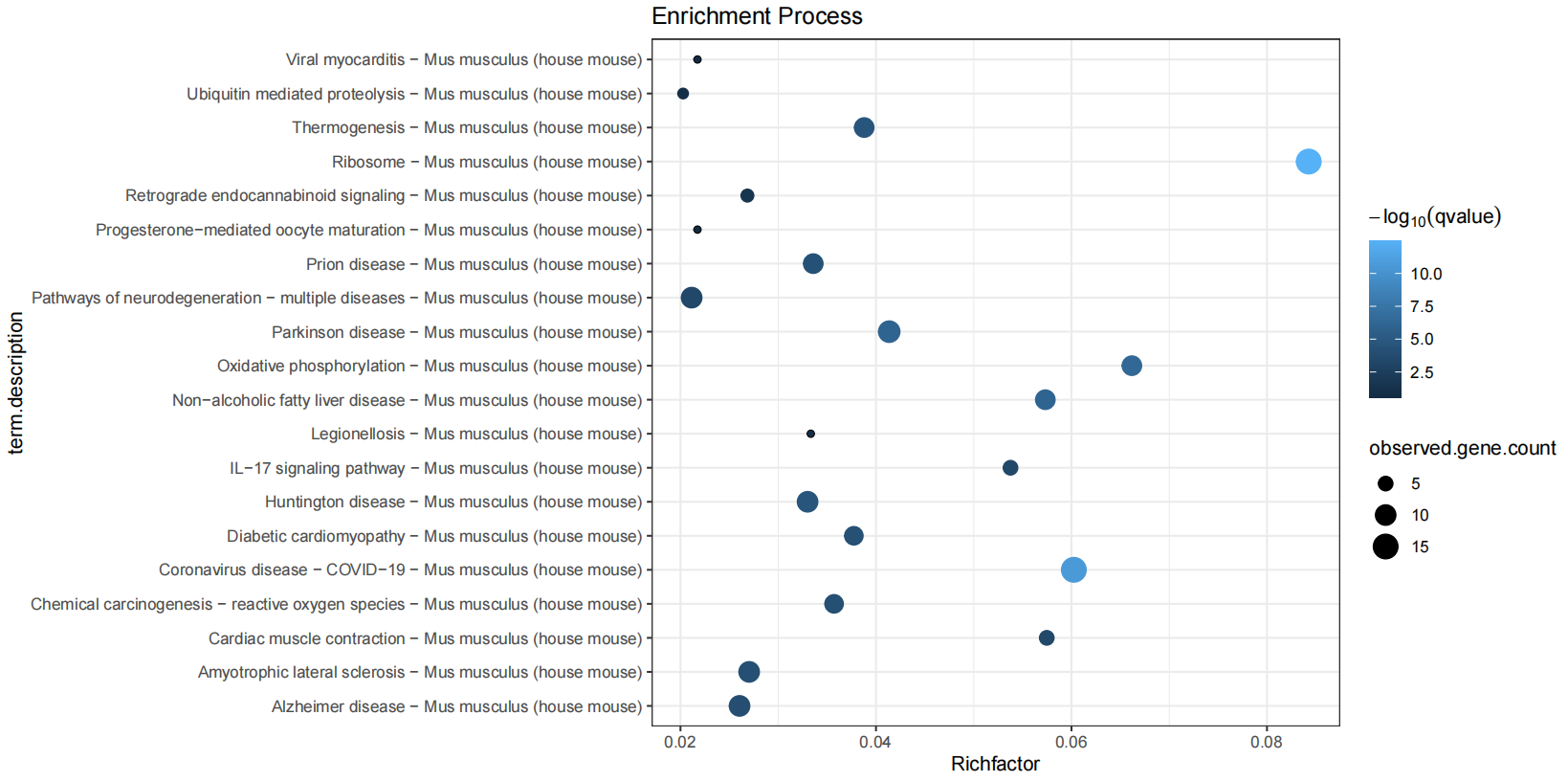


**Figure S5. Analysis of KEGG enrichment of different genes in M2 macrophages.**


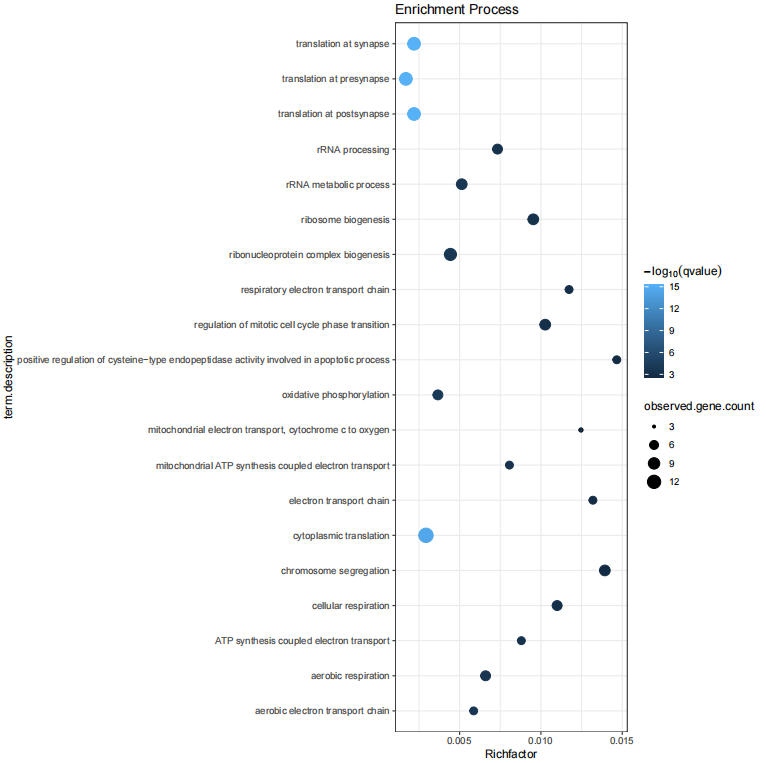


**Figure S6. GO enrichment of different genes in M2 macrophages was analyzed in BP group.**


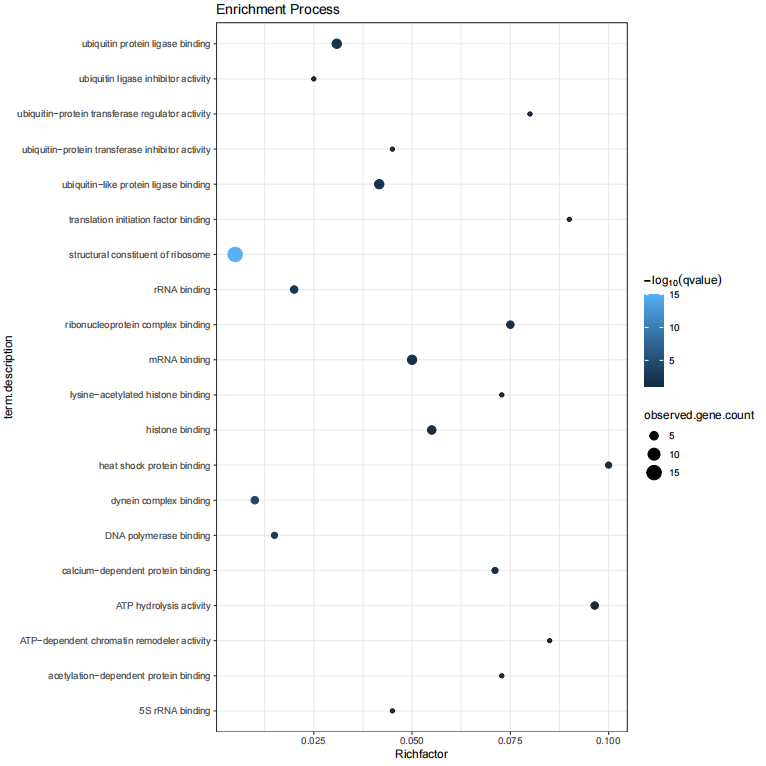


**Figure S7. GO enrichment analysis of different genes in M2 macrophages in MF group.**


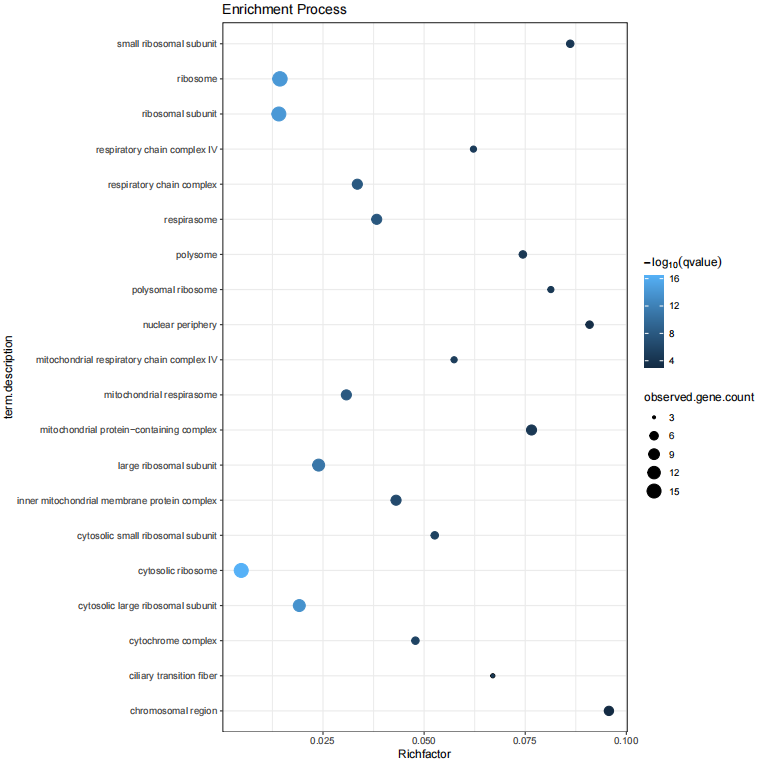


**Figure S8. GO enrichment of different genes in M2 macrophages was analyzed in CC group.**
