# Supplementary material for: Mechanism exploration of synergistic photo-immunotherapy strategy based on a novel exosome-like nanosystem for remodeling the immune microenvironment of HCC
Source: Nano Converg. 2024 Aug 14;11:31. doi: 10.1186/s40580-024-00441-6 (PMC11324638; doi:10.1186/s40580-024-00441-6)
Supplement: Supplementary file 2 — Supplementary Material 2 [file 40580_2024_441_MOESM2_ESM.pdf]

哈尔滨医科大学医学伦理审查申请表

项目名称	富氧纳米微泡介导声动力疗法增强铁死亡抗肝癌的实验研究		
申报课题来源	国家自然科学基金面上项目		
项目负责人	程文	职称	主任医师
电子信箱	chengwen@hrbmu.edu.cn	电话	13313677182
所在单位	哈尔滨医科大学附属肿瘤医院	科室	超声科
研究材料	☑永生细胞系 ☑动物 ☑人病史数据 □人生物标本		
材料来源	☑购买 □以往临床采集保存 ☑研究采集 □去标识后获取		

单位伦理委员会意见：

哈尔滨医科大学附属肿瘤医院伦理委员会的组成、职责及工作程序严格遵循国际伦理准则、GCP 规范及中国相关的法律法规。

哈尔滨医科大学附属肿瘤医院伦理委员会对此申请项目意见：**同意该项目申报。项目立项后，需通过伦理委员会审查批准，方可开展实施研究。**

伦理委员会主任委员：赵宏印长　　单位伦理委员会公章：哈尔滨医科大学附属肿瘤医院伦理委员会

2021 年 2月 21日
